# The Entrapment and Concentration of SARS-CoV-2 Particles with Graphene Oxide: An In Vitro Assay

**DOI:** 10.3390/nano13020343

**Published:** 2023-01-14

**Authors:** Beatriz Parra, Adolfo Contreras, José Herminsul Mina, Mayra Eliana Valencia, Carlos David Grande-Tovar, Carlos Humberto Valencia, Cristina Ramírez, Germán Armando Bolívar

**Affiliations:** 1 Grupo de Virus Emergentes y Enfermedad (VIREM), Departamento de Microbiología, Facultad de Salud, Universidad del Valle, Calle 4B No. 36-00, Santiago de Cali 760032, Colombia; 2 Grupo Medicina Periodontal, Escuela de Odontología, Facultad de Salud, Universidad del Valle, Calle 4B No. 36-00, Santiago de Cali 760043, Colombia; 3 Grupo Materiales Compuestos (GMC), Escuela de Ingeniería de Materiales, Facultad de Ingeniería, Universidad del Valle, Calle 13 No. 100-00, Santiago de Cali 760032, Colombia; 4 Grupo de Investigación de Fotoquímica y Fotobiología, Facultad de Ciencias, Universidad del Atlántico, Carrera 30 Número 8-49, Puerto Colombia 081008, Colombia; 5 Grupo Biomateriales Dentales, Escuela de Odontología, Universidad del Valle, Calle 4B No. 36-00, Santiago de Cali 76001, Colombia; 6 Grupo de Investigación en Ingeniería de Procesos Agroalimentarios y Biotecnológicos (GIPAB), Escuela de Ingeniería de Alimentos, Facultad de Ingeniería, Universidad del Valle, Calle 13 No. 100-00, Santiago de Cali 760032, Colombia; 7 Grupo de Investigación en Microbiología y Biotecnología Aplicada (MIBIA), Departamento de Biología, Facultad de Ciencias Naturales y Exactas, Universidad del Valle, Calle 13 No. 100-00, Santiago de Cali 760032, Colombia

**Keywords:** SARS-CoV-2 virus, virus entrapment, graphene oxide, viral plaques, VERO cells

## Abstract

Previous studies have suggested that graphene oxide (GO) has some antiviral capacity against some enveloped viruses, including SARS-CoV-2. Given this background, we wanted to test the in vitro antiviral ability to GO using the viral plaque assay technique. Two-dimensional graphene oxide (GO) nanoparticles were synthesized using the modified Hummers method, varying the oxidation conditions to achieve nanoparticles between 390 and 718 nm. The antiviral activity of GO was evaluated by experimental infection and plaque formation units assay of the SARS-CoV-2 virus in VERO cells using a titrated viral clinical isolate. It was found that GO at concentrations of 400 µg/mL, 100 µg/mL, 40 µg/mL, and 4 µg/mL was not toxic to cell culture and also did not inhibit the infection of VERO cells by SARS-CoV-2. However, it was evident that GO generated a novel virus entrapment phenomenon directly proportional to its concentration in the suspension. Similarly, this effect of GO was maintained in assays performed with the Zika virus. A new application for GO nanoparticles is proposed as part of a system to trap viruses in surgical mask filters, air conditioning equipment filters, and air purifier filters, complemented with the use of viricidal agents that can destroy the trapped viruses, an application of broad interest for human beings.

## 1. Introduction

The pandemic caused by the acute respiratory failure virus SARS-CoV-2 generated significant changes in the behaviour and conduct of human beings. Social distancing, maintaining good hand hygiene, and using face masks have been some of the preventive measures for life protection recommended by the World Health Organization [1] to mitigate the high infection rates before and after the development of vaccines. Nanomaterials with antiviral capacities, such as CuO, Ag, and TiO_2_, among others [2], have been proposed to be incorporated into face masks and other personal protective equipment (PPE) to increase filtration efficiency and minimize infection risks among healthcare personnel and populations [3]. Graphene oxide (GO) consists of a graphene sheet with oxygen-rich functionalities randomly distributed on its surface [4] that has shown antibacterial and antiviral properties [5,6,7,8,9,10,11] thanks to its ability to interact with microorganisms through hydrogen bridges, e.g., via electrolytic interactions, electrostatic trapping, the inactivation of virus and the host cell receptor, the physical–chemical destruction of viral species, and redox reactions, or via a mechanical shearing effect by breaking the virus envelope, so it has been proposed as a single or combined antiviral agent to be incorporated in PPE. The entrapment phenomenon exhibited by graphene materials has been studied mainly in bacteria [8,10,11,12,13], among which Gram-negative *E. coli* and Gram-positive *S. aureus* stand out, suggesting that they are capable of enveloping external substances and entrapping particles by their oxygenated functional groups due to their highly flexible structure [12]. Akhavan et al. [13] were able to entrap *E. coli* in graphene oxide sheets and observed that when released by sonication, these bacteria could interact again with their environment, consume glucose, and grow, so they proceeded to treat them by near-infrared irradiation (at 808 nm) and concluded that GO nanomaterials could be used as encapsulants for trapping bacteria later inactivated by subsequent antimicrobial processes [14].

Cheng [7] et al. first reported the antiviral activity of GO and GO-Ag against enveloped and non-enveloped viruses. The antiviral mechanisms of GO are mainly based on the blocking of viral entries and the interference with viral membrane fusion within a partial inhibition ability (25%) of GO-Ag (1 mg/mL) against coronavirus (47,000 TCID_50_/mL) as compared to an almost complete or 97% inhibition against influenza virus (100 TCID_50_/mL) by Ag NPs (0.05 mg/mL) in Xiang’s study [8]. Thus, it is crucial to consider that GO sheets showed some antiviral activity against coronavirus. Future studies should also consider a summative and synergistic effect from GO sheets plus metallic particles against the enveloped virus.

Some encouraging results have been found on the use of GO nanoparticles to combat the pandemic generated by SARS-CoV-2 and its use for the development of PPE. These articles [6,15,16] highlight the ability of GO to inactivate the virus by using centrifugated GO and SARS-CoV-2 infectivity on VERO cells and suggesting an antiviral effect since these supernatants were possibly virus-free, which might be partially true because the SARS-CoV-2 was entrapped and precipitated by GO after centrifugation. Therefore, this study aimed to confirm the previously described antiviral capacity of GO against SARS-CoV-2 or to discern this GO-viral entrapment ability by using an infection model of SARS-CoV-2 on VERO cells under in vitro conditions. The hypothesis to be tested is that GO does not present a direct antiviral activity against SARS-CoV-2 but an intense entrapment activity. The novelty of this study is to reveal the actual effect of GO on SARS-CoV-2 in infectivity in vitro cellular model. 

## 2. Materials and Methods

### 2.1. Materials

For the synthesis of GO, flake graphite (325 mesh, Alfa Aesar, Tewksbury, MA, USA), sulfuric acid (H_2_SO_4_), potassium permanganate (KMnO_4_), hydrogen peroxide (H_2_O_2_), and 2-propanol from Merck (Burlington, MA, USA) were used. The cell line employed in this research was ATCC CCL-81 and Dulbecco’s modified Eagle cell culture media, inactivated bovine fetal serum, glutamine, streptomycin, and penicillin. All reagents used in the investigation were employed as received without further modification.

### 2.2. Graphene Oxide Synthesis

For the synthesis of GO nanoparticles, the modified Hummers’ method reported by Valencia et al. was followed, with some variations [4]. Briefly, 3 g of graphite was added to 400 mL of H_2_SO_4_ and mixed with different ratios of KMnO_4_. Three types of GOs, named GO 1 to GO S2, were developed. For the GO 1 sample, 3 g of KMnO_4_ was added every 24 h for three days, while for GO 2 and GO S2 samples, 2 g additions of KMnO_4_ were added every 24 h for three days. The oxidation reaction of graphite was stopped 24 h after adding the last amount of KMnO_4_, incorporating half of the mixture in 500 mL of ice water and subsequently 3 mL of H_2_O_2_. Finally, washes were performed with type I water and isopropanol until a pH higher than 5 was obtained.

### 2.3. Physicochemical Characterization of Graphene Oxide

GO was characterized by performing physicochemical (FTIR, particle size) and thermal (TGA) tests. In this sense, FTIR was performed in an IR Affinity-1 infrared spectrophotometer (Shimadzu, Kyoto, Japan) in the range of 500–4000 cm^−1^ in transmittance mode using the attenuated total reflectance (ATR) method. The TGA was carried out on a TGA Q50, utilizing a heating ramp of 20 °C/min, from 25 to 800 °C, using air as the test gas. Finally, the particle size was determined on a Zetasizer Nano ZS DLS (Malvern Panalytical, Jarman Way, Royston, UK).

### 2.4. Evaluation of the Antiviral Activity of GO

The inhibition of or reduction in viral plaque formation in VERO cells by PRNT90 (90% Plaque Reduction Neutralization assay) was used to evaluate the viricidal or antiviral activity of GO, employing a clinical isolate of titrated SARS-CoV-2 [17]. VERO cells monolayers from African green monkey ATCC CCL-81 were cultured with Dulbecco’s modified Eagle medium (DMEM), supplemented with 10% inactivated fetal bovine serum (FBS), 1 mM glutamine, 1% streptomycin, and penicillin, and incubated with a 5% CO_2_ atmosphere at 37 °C. Twenty-four-well culture plates were prepared by washing with PBS, and then cells were trypsinized, counted, and seeded in cell culture microplates at a concentration of 6 × 10^4^ cells/mL.

**Figure 1:**
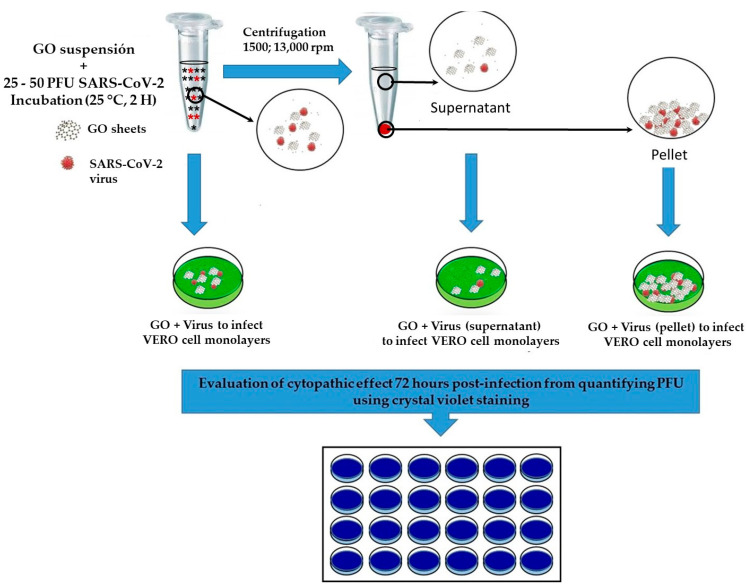
Schematic evaluation of GO antiviral activity by reducing viral plaque formation in VERO cells.

The effects of GO against SARS-CoV-2 were performed by inhibiting the plaque-forming unit (PFU). Various suspensions from GO 1 to GO S2 diluted in sterile saline solution were used, as shown in Table 1. A 0.4 mg/mL concentration of GO was incubated with the same volume of a SARS-CoV-2 suspension producing approximately 250–500 PFU per mL in VERO cells, and incubation was performed at room temperature for 2 h under agitation. Subsequently, 0.2 mL of the GO + virus mixture was used to infect VERO cells monolayers per well in a 24 well plating assay. Simultaneously, another volume of the same pre-incubated GO + virus mixture was centrifuged at 13,000 rpm for 5–7 min, and the supernatant was used to infect VERO cells in the plating assay. The GO-containing pellet was also employed to infect another separate well in the same plating assay. The cytopathic effect was observed 72 h post-infection, which involved quantifying PFU using crystal violet staining. In all plating assays, VERO cells without any treatment, SARS-CoV-2 virus plaque controls without GO treatment, and controls with GO but without the virus were used as negative controls to determine the self-toxicity of GO on the cell cultures. SARS-CoV-2 virus manipulation was performed with type III biosafety standards. Figure 1 shows a scheme summarizing the methodology followed in the antiviral evaluation. 

The nomenclature used for the GO solutions to evaluate antiviral activity is shown in Table 1. Assays associated with the biocompatibility of graphene oxide were performed at increasing concentrations of GO from 50 nanograms of GO to 0.4 mg/mL.

## 3. Results and Discussion

### 3.1. GO Characterization

#### 3.1.1. Fourier Transform Infrared Spectroscopy (FTIR)

**Figure 2:**
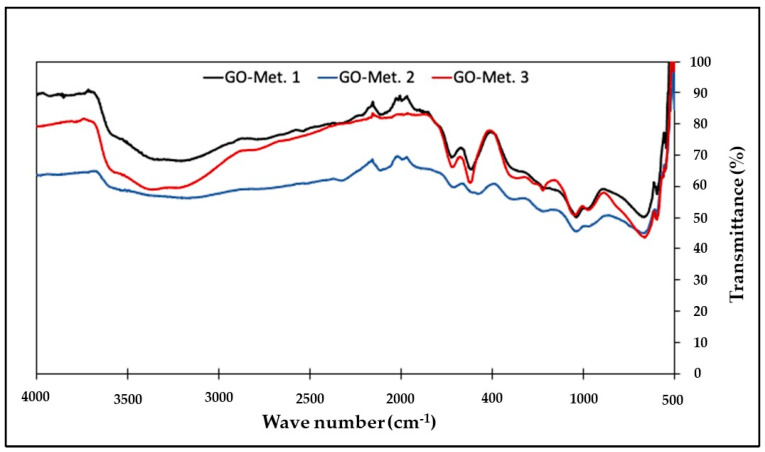
FTIR spectrum of the GO samples analysed.

Characteristic bands of GO were found in the three samples studied. These distinctive bands were observed at 3346 cm^−1^ for the O-H tensile stretching, at 1720 cm^−1^ for C=O bonds, at 1620 cm^−1^ for aromatic C=C, at 1365 cm^−1^ for the C-O-H bond, at 1200 cm^−1^ for the C-O bond stretching, and at 1042 cm^−1^ for the C-O-C bond, as Figure 2 shows the FTIR spectra of the GO samples synthesized and used in this investigation. These signals are similar to those reported in other studies [18,19].

#### 3.1.2. Thermogravimetric Analysis (TGA)

**Figure 3:**
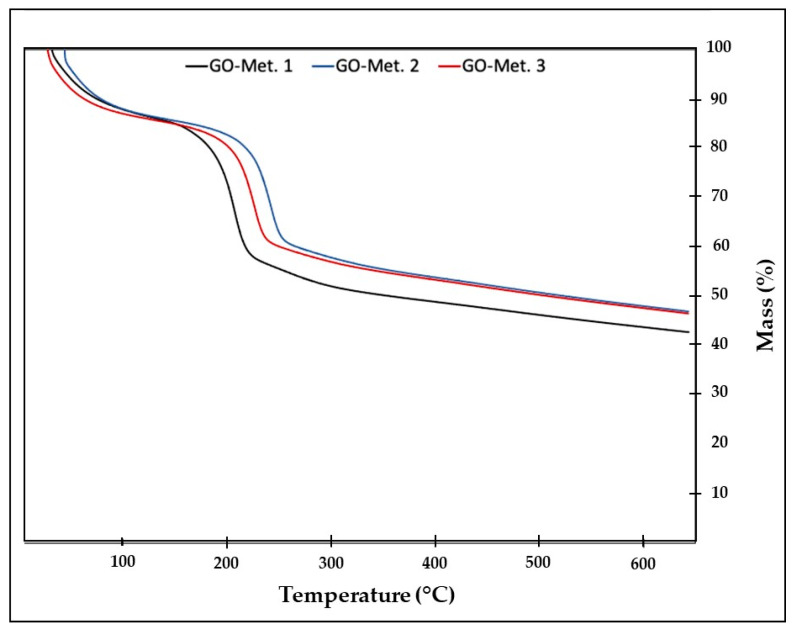
Thermogravimetric analysis curves of analysed GO samples.

This technique was used to recognize changes in the material after oxidation, i.e., to verify the presence of functional groups in the material qualitatively. Figure 3 records the results of the analysis for the GO samples. Generally, three GO degradation steps were found to occur at three main temperatures (<100 °C, 200 °C, and above 250 °C). The mass loss of GO for temperatures below 100 °C is associated with eliminating adsorbed or adsorbed water molecules; for temperatures around 200 °C, the loss of oxy-generated functional groups is generated, and at higher temperatures, the decomposition of leading carbon chains is generated [20].

It should be noted that the GO obtained by method 1 presented the lowest thermal stability since it is the one with the highest mass loss, so it is expected that this result is directly related to the change in the sample’s degree of oxidation.

#### 3.1.3. Dynamic Light Scattering (DLS)

The literature shows that the material’s morphology, dimension, and transparency depend significantly on oxidation level and exfoliation strategy [21]. Table 2 shows the different particle sizes of the synthesized samples, where a notable difference can be seen in the GO 2 sample concerning the others, since this is the one with the largest particle size. Several studies where different degrees of oxidation have been obtained show that the more oxygenated groups are present in the structure, with smaller particle sizes being associated with the degree of oxidation [22,23]. On the other hand, it has been observed that the films show a highly transparent or less rough morphology as the oxidation degree increases, decreasing the number of layers present, which favours the exfoliation process, allowing a smaller particle size [23,24]. However, for the moment, particle sizes outside the range were considered optimal for passive tumour accumulation (100 to 200 nm) [25,26].

### 3.2. Evaluation of Antiviral Activity

**Figure 4:**
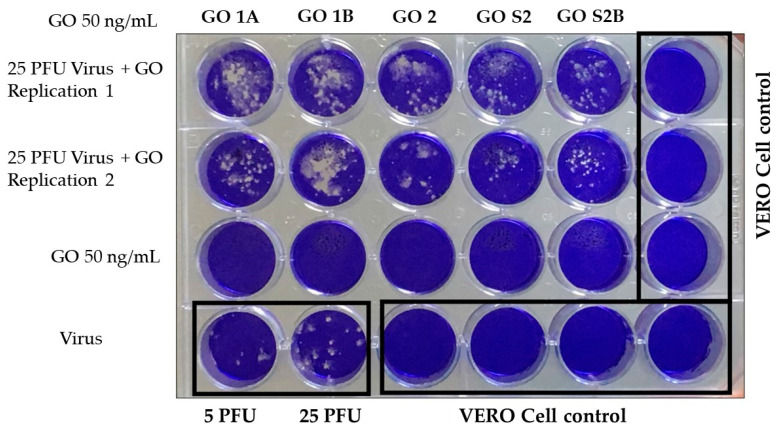
Assay of the antiviral activity of GO 50 ng/mL against SARS-CoV-2 in VERO cells by plating.

An evaluation of the antiviral activity against SARS-CoV-2 of GO particles previously dispersed in a suspension of fetal serum (FS) was initiated from a viral plaque reduction assay in VERO cells. Here, we worked with 25 plaque-forming units (PFUs) of virus plus GO and performed each assay in the duplication and control of VERO cells without including GO or virus, as illustrated in Figure 4. 

It was found that GO at a concentration of 50 ng/mL during a 30-min exposure did not show toxicity to VERO cells; this result is significant given that there are reports which indicate that there are functionalized graphene oxide sheets that are recommended for non-medical use in light of the specific cytotoxicity values evaluated with L-929 mouse fibroblast cells using the MTT assay [12]. The OG used in none of the formulations inhibited the infection of VERO cells by SARS-CoV-2 since, in all cases, plaque-forming units were generated.

**Figure 5:**
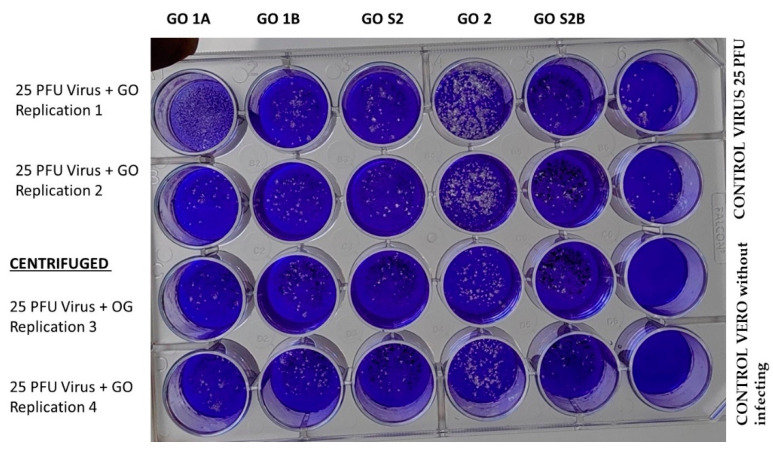
SARS-CoV-2 virus plating assay in VERO cells using 400 µg/mL of the centrifuged and non-centrifuged OG, OG, + virus blend.

Subsequently, another type of experiment was proposed, where the concentration of GO was increased to 400 µg/mL (undiluted), maintaining the 25 PFU of virus in 100 uL of medium + 100 uL GO in SF (400 µg/mL). The system was incubated for 2 h at room temperature, and the plating assay was performed on VERO cells (centrifuging × 7 min at 1500 rpm and without centrifuging the mixture of GO + virus). Figure 5 shows the results, where it can be observed that under the conditions tested, GO had no virucidal effect; it is evident that GO precipitates with the virus or “entraps or agglutinates” the virus and causes precipitation of it with the sediment and higher amount of viral PFU compared with the 25 PFU of SARS-CoV-2 control wells. This GO capture reduced the viral infectivity in the supernatants of this in vitro live virus model of SARS-CoV-2 infection. The entrapment capacity of graphene oxide has mainly been studied in bacteria, attributing this phenomenon to the flexibility of the films and their ability to wrap substances and retain them with their oxygenated functional groups [8,10,11,12,13]. In the case of SARS-CoV-2, De Maio et al. [15] reported behaviour similar to that found in the present investigation, where the virus was efficiently retained in textile fibres previously treated with graphene oxide. It is noteworthy that GO 2 appears to have a virus-concentrating effect since more virus plaques are seen. Likewise, centrifugation affects only GO 2, reducing the amount of “entrapped” viruses in the supernatants but not in the sedimented pellets.

**Figure 6:**
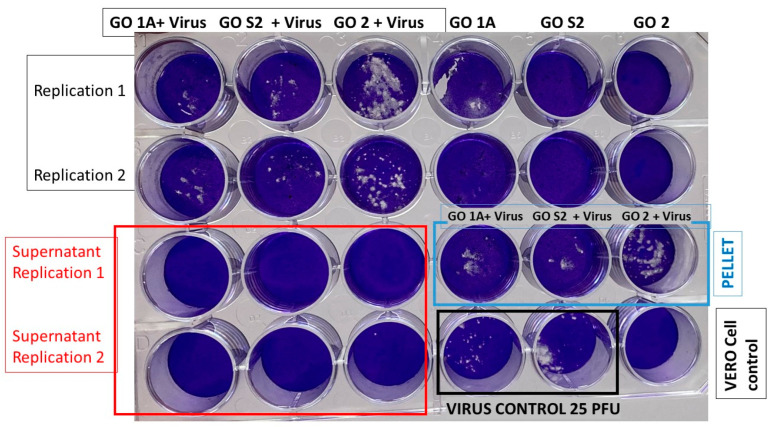
SARS-CoV-2 virus plating assay in VERO cells at concentrations of 400 µg/mL of centrifuged and non-centrifuged OG alongside the OG + virus mixture at a centrifugation speed of 13,000 rpm.

To confirm the entrapment phenomenon, the previous experiment was repeated for the samples of GO 1A, S2, and 2, increasing the centrifugation speed from 1500 to 13,000 rpm for a time of 5 min by inoculating the supernatant and the centrifuged pellet. Figure 6 shows the results of the test. Here, it is confirmed that GO precipitates or entraps the virus, and there is a more significant entrapment effect when the synthesis method corresponding to GO 2 is used since more virus plaques are generated.

**Figure 7:**
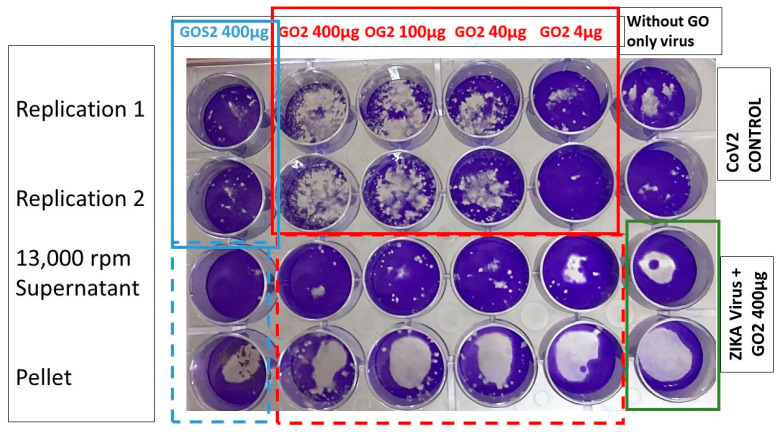
SARS-CoV-2 virus and Zika virus plating assay in VERO cells at concentrations ranging from 400 to 4 µg/mL of centrifuged OGS2 (pellet) and supernatant in an OG + virus mixture at a centrifugation speed of 13,000 rpm.

After confirming the virus entrapment effect presented by GO, the dose response of the GO 2 sample was tested, varying the concentration in values of 400 µg/mL, 100 µg/mL, 40 µg/mL, and 4 µg/mL, on a fixed dose of virus. Figure 7 shows the images obtained, where additionally, for comparison purposes, the zika virus is included. It was determined that the GO 2 synthesis method is the most effective for capturing the virus, in addition to the fact that there is a dose response of GO 2; the higher the concentration of GO, the more that the capacity to capture the virus increases and decreases with lower concentrations of GO. The effective range goes from 400 µg/mL to 40 µg/mL, while a concentration of 4 µg/mL did not have these effects. Likewise, upon centrifugation at 13,000 rpm, the supernatant loses infectivity compared to the GO2 + virus mixture without centrifugation, while the pellet continues to be infective. 

## 4. Conclusions

It was possible to evaluate the viricidal or antiviral activity of GO against titrated SARS-CoV-2 by using a viral plaque assay on VERO cells directly and also to discern the effect of centrifuging a suspension of GO virus to evaluate both the infectivity of supernatant and the infectivity of pellet. 

It was discovered that GO generates an entrapment–precipitation effect of the SARS-CoV-2 virus, which increases when the nanomaterial concentration is also increased, as the synthesis method of the GO 2 sample is the most effective in terms of the virus entrapment phenomenon, compared to the other GO samples tested here. 

This viral entrapment effect could also be evidenced in the case of the use of the Zika virus, maintaining a similar trend to that obtained with SARS-CoV-2, so it does not seem to be a virally species-associated phenomenon. 

Similarly, it was observed that there is a dose response associated with the GO 2 sample for SARS-CoV-2 virus trapping. The higher the concentration of GO 2, the more capture activity of the virus, and it decreases for a lower concentration of GO, being effective from 400 to 40 µg/mL. A concentration of 4 µg/mL cannot trap any virus.

This study confirmed a viral entrapment effect of GO over SARS-CoV-2 and on Zika viruses. In this sense, it was clear that the loss of infectivity in the supernatant is not due to a viricidal effect of GO, but due to the fact that this zone lacks the virus because it is trapped in the pellet.

Although it was demonstrated that GO does not have viricidal capacity against SARS-CoV-2 and Zika, the nanomaterial can be studied in other forms of application, in which it is exposed on surfaces to preserve its “trapping” or concentrating potential (surgical mask filters, filters in air conditioning equipment, and air purifier filters, among others). Likewise, viricidal agents and photothermal processes can inactivate trapped viruses.

## Figures and Tables

**Table 1:** Description of the GO samples studied.

Designation	Description
GO 1	GO synthesized with the addition of 3 g of KMnO_4_ every 24 h.
GO 2	GO synthesized with the addition of 2 g of KMnO_4_ every 24 h.
GO S2	GO synthesized from the supernatant of GO 2 synthesis.

**Table 2:** Particle size results from dynamic light scattering (DLS).

Particle Size—nm (Standard Deviation)		
**GO 1**	GO 2	GO S2
582.5 (58.14)	718.0 (53.49)	390.3 (36.42)

## Data Availability

The raw/processed data required to reproduce these findings cannot be shared as the data also form part of an ongoing study.

## References

[1] World Health Organization (2021). Advice for the Public: Coronavirus Disease (COVID-19). https://www.who.int/emergencies/diseases/novel-coronavirus-2019/advice-for-public.

[2] El-Atab N., Mishra R.B., Hussain M.M. (2021). Toward nanotechnology-enabled face masks against SARS-CoV-2 and pandemic respiratory diseases. Nanotechnology.

[3] El-Atab N., Qaiser N., Badghaish H., Shaikh S.F., Hussain M.M. (2020). Flexible Nanoporous Template for the Design and Development of Reusable Anti-COVID-19 Hydrophobic Face Masks. ACS Nano.

[4] Valencia-Zapata M.E., Ruiz L.M., Mina-Hernandez J.H., Delgado-Ospina J., Grande-Tovar C.D. (2020). Acrylic Bone Cements Modified with Graphene Oxide: Mechanical, Physical, and Antibacterial Properties. Polymers.

[5] Ye S., Shao K., Li Z., Guo N., Zuo Y., Li Q., Lu Z., Chen L., He Q., Han H. (2015). Antiviral Activity of Graphene Oxide: How Sharp Edged Structure and Charge Matter. ACS Appl. Mater. Interfaces.

[6] Thakur A.K., Sathyamurthy R., Ramalingam V., Lynch I., Sharshir S.W., Ma Z., Poongavanam G., Lee S., Jeong Y., Hwang J.Y. (2021). A case study of SARS-CoV-2 transmission behavior in a severely air-polluted city (Delhi, India) and the potential usage of graphene based materials for filtering air-pollutants and controlling/monitoring the COVID-19 pandemic. Environ. Sci. Process. Impacts.

[7] Chen Y.N., Hsueh Y.H., Hsieh C.T., Tzou D.Y., Chang P.L. (2016). Antiviral Activity of Graphene-Silver Nanocomposites against Non-Enveloped and Enveloped Viruses. Int. J. Environ. Res. Public Health.

[8] Seifi T., Kamali A.R. (2021). Anti-pathogenic activity of graphene nanomaterials: A review. Colloids Surf. B Biointerfaces.

[9] Seifi T., Kamali A.R. (2021). Antiviral performance of graphene-based materials with emphasis on COVID-19: A review. Med. Drug Discov..

[10] Mangadlao J.D., Santos C.M., Felipe M.J.L., De Leon A.C.C., Rodrigues D.F., Advincula R.C. (2015). On the Antibacterial Mechanism of Graphene Oxide (GO) Langmuir-Blodgett Films. ChemComm.

[11] Wang X., Lu P., Li Y., Xiao H., Liu X. (2016). Antibacterial activities and mechanisms of fluorinated graphene and guanidine-modified graphene. RSC Adv..

[12] He G.-Y., Dai W., Zhao Y.-T., Chen Q., Sun X.-Q., Chen H.-Q., Wang X. (2014). A facile synthesis of Ag@graphene-nanosheet composite with enhanced antibacterial activity and acceptable environmental safety. Monatsh. Chem..

[13] Akhavan O., Ghaderi E., Esfandiar A. (2011). Wrapping Bacteria by Graphene Nanosheets for Isolation from Environment, Reactivation by Sonication, and Inactivation by Near-Infrared Irradiation. J. Phys. Chem. B.

[14] Xiang D.X., Chen Q., Pang L., Zheng C.L. (2011). Inhibitory effects of silver nanoparticles on H1N1 influenza A virus in vitro. J. Virol. Methods.

[15] De Maio F., Palmieri V., Babini G., Soon-Shiong P., Sali M., Papi M., Spilman P. (2021). Graphene nanoplatelet and graphene oxide functionalization of face mask materials inhibits infectivity of trapped SARS-CoV-2. IScience.

[16] Figerez S.P., Patra S., Rajalakshmi G., Narayanan T.N. (2020). Graphene oxide-based rechargeable respiratory masks. Oxf. Open Mater. Sci..

[17] Lopez-Alvarez D., Parra B., Cuellar W.J. (2020). Genome Sequence of SARS-CoV-2 Isolate Cali-01, from Colombia, Obtained Using Oxford Nanopore MinION sequencing. Microbiol Resour Announc.

[18] Tabatabaee S., Baheiraei N., Salehnia M. (2022). Fabrication and characterization of PHEMA–gelatin scaffold enriched with graphene oxide for bone tissue engineering. J. Orthop. Surg. Res.

[19] Valencia C., Valencia C.H., Zuluaga F., Valencia M.E., Mina J.H., Grande-Tovar C.D. (2018). Synthesis and Application of Scaffolds of Chitosan-Graphene Oxide by the Freeze-Drying Method for Tissue Regeneration. Molecules.

[20] Toh G.Y., Ong H.L., Lim H.N., Huang N.M., Akil H.M., Villagracia A.R., Nonato G., Santos C., Lee H.L. (2017). Tailoring the chemical and structural properties of graphene oxide nanoplatelets synthesized at room temperature with different processing times. J. Phys. Sci..

[21] Krishnamoorthy K., Veerapandian M., Yun K., Kim S.J. (2013). The chemical and structural analysis of graphene oxide with different degrees of oxidation. Carbon.

[22] Ma L., Lyu S.S., Dai Y., Pei X.Y., Mo D.C., Fu Y.X. (2019). Lithium storage properties of NiO/reduced graphene oxide composites derived from different oxidation degrees of graphite oxide. J. Alloys Compd..

[23] Liu X., Huang Y., Duan S.h., Wang Y., Li J., Chen Y., Hayat T., Wang X. (2016). Graphene oxides with different oxidation degrees for Co(II) ion pollution management. Chem. Eng. J..

[24] Al-Gaashani R., Najjar A., Zakaria Y., Mansour S., Atieh M.A. (2019). XPS and structural studies of high quality graphene oxide and reduced graphene oxide prepared by different chemical oxidation methods. Ceram. Int..

[25] De Melo-Diogo D., Pais-Silva C., Dias D.R., Moreira A.F., Correia I.J. (2017). Strategies to Improve Cancer Photothermal Therapy Mediated by Nanomaterials. Adv. Healthc. Mater..

[26] De Melo-Diogo D., Costa E.C., Alves C.G., Lima-Sousa R., Ferreira P., Louro R.O., Correia I. (2018). POxylated Graphene Oxide Nanomaterials for Combination Chemo-Phototherapy of Breast Cancer Cells. Eur. J. Pharm. Biopharm..

